# Individual and community-level determinants of poor tetanus toxoid immunization among pregnant women in Ethiopia using data from 2016 Ethiopian demographic and health survey; multilevel analysis

**DOI:** 10.1186/s13690-021-00622-3

**Published:** 2021-06-04

**Authors:** Alemneh Mekuriaw Liyew, Hiwotie Getaneh Ayalew

**Affiliations:** 1 Department of Epidemiology and Biostatistics, Institute of Public Health, College of Medicine and Health Sciences and comprehensive specialized hospital, University of Gondar, Gondar, Ethiopia; 2 Department of midwifery, school of nursing and midwifery, college of medicine and health sciences, Wollo University, Dessie, Ethiopia

**Keywords:** Tetanus toxoid immunization, Pregnancy, Multi-level analysis, Ethiopia

## Abstract

**Background:**

Tetanus is a vaccine-preventable disease that can occur in all populations, with neonates and pregnant women being at the most risk. Ethiopia has the highest maternal and neonatal tetanus morbidity and mortality rates. Besides, only 49% of mothers get vaccinated with adequate tetanus toxoid in Ethiopia which is below the world health organization recommendation. To date, there is limited evidence on the individual and community level determinants of poor tetanus toxoid (TT) immunization. Therefore, this study aimed to assess individual and community-level factors associated with poor TT immunization coverage in Ethiopia.

**Method:**

Secondary data analysis was conducted using the 2016 Ethiopian demographic and health survey. A total of 7043 pregnant women were included in the current study. A multilevel logistic regression model was used to identify individual and community level determinants of poor tetanus toxoid immunization. Finally, the adjusted odds ratio with a 95% confidence interval was reported.

**Results:**

In the multilevel logistic regression model adjustment**,** having no Antenatal care visit (AOR = 5.64; 95% CI:2.48,7.30) and having one to three antenatal care visit (AOR = 1.50; 95% CI: 1.19–1.82); poor wealth index (AOR = 1.26; 95% CI: 1.03, 1.54); not being exposed to media (AOR = 1.29; 95% CI: 1.10, 1.51); maternal unemployment (AOR = 1.15; 95% CI: 1.10, 1.31); rural residence (AOR = 1.13; 95% CI: 1.08, 1.72); and high community illiteracy (AOR = 1.28; 95% CI: 1.03, 1.58) were associated with higher odds of poor tetanus toxoid immunization. Whereas, iron uptake during pregnancy (AOR = 0.59; 95% CI: 0.51, 0.68), was associated with lower odds of poor tetanus toxoid immunization.

**Conclusion:**

In this study tetanus toxoid (TT) vaccine utilization was affected by both community and individual-level factors. Therefore, focusing on antenatal care services especially encouraging pregnant women to have at least four visits, consulting women to be exposed to media, improving community literacy and maternal employment will help to minimize TT underutilization.

## Background

Tetanus is a life-threatening bacterial disease caused by spores of Clostridium tetani found throughout the world [1]. It is common in areas of poor hygiene, the most disadvantaged, economically poor, and without access to adequate health services [2]. Although tetanus can occur in all age groups, neonates, and women with recent pregnancies are most at risk, especially when childbirths take place under unhygienic conditions [3].

Case fatality rates from tetanus in resource-limited settings can be up to 100%, though with adequate medical care it can be reduced to 10–20% [4]. Estimates indicate that in 2004 tetanus caused 128,250 newborns and 30,000 maternal deaths, mainly in Africa and Asia [5]. Even though there is a great stride in the reduction of maternal and neonatal mortality through maternal and neonatal tetanus elimination initiatives, Ethiopia is among 21 countries that had not yet attained the elimination of the disease [6].

Prevention of maternal and neonatal tetanus can be achieved through simple hygienic birth-related practices and tetanus toxoid vaccination of mothers [7, 8]. This is strengthened by the evidence from a systematic review that demonstrates the TT immunization of women of childbearing age reduces the risk of neonatal tetanus [9]. Besides, a meta-analytic study shows that TT immunization of pregnant women with at least two doses (TT2) can decrease neonatal mortality due to tetanus by 94% [10]. Therefore, the World Health Organization (WHO) recommends a total of five doses of TT vaccine for women with no history of tetanus toxoid (TT) immunization. The first two doses are given in the first trimester, each within a range of 1 month, and another dose during the next pregnancy or within a 1-year interval.

Globally, 40 million pregnant mothers remained in need of immunization against maternal tetanus. The number of mothers living in high-risk areas and protected with at least two doses of TT vaccination during the 1999–2006 supplementary immunization activities was only 73 million [11]. Currently, TT2+ immunization coverage among pregnant mothers accounts for 75% worldwide ranging from 95% in South East Asia to 53% in the East Mediterranean and 63% in Africa [12].

Ethiopia has the highest maternal and neonatal tetanus morbidity and mortality rates in the world due to low TT immunization coverage. The Ethiopian Demographic and Health Survey (EDHS) report of 2011 indicated that only 48% [13] of mothers were protected against tetanus at birth [13]. Very static progress had been shown up to 2016 as evidenced by only 49% of mothers were protected at birth [14]. Despite the country’s effort of interventional policy to meet the WHO goals towards Maternal and Newborn Tetanus Elimination (MNTE) through extended immunizations and campaigning tetanus toxoid vaccinations, Ethiopia continues to have the higher tetanus morbidity and mortality rates [14].

Poor access to vaccines and lack of knowledge are primary factors for poor vaccination coverage in Bangladesh [15]. Other reasons for low coverage include lack of knowledge of time and place for TT immunization and misconceptions of vaccines as contraceptive agents [16]. Moreover, different sociodemographic factors such as antenatal care visit [17, 18], wealth index [17, 19], women’s employment [17], maternal age at delivery, maternal education [19, 20], place of residence [19, 21], multiparity [21], health care decision making, contraceptive use and husband education [19] have an association with tetanus toxoid immunization.

In Ethiopia, though the 2016 Demographic and Health Survey (EDHS) demonstrated the under-utilization of TT immunization during pregnancy [14], to date there is limited evidence on the contributing factors to this poor immunization. Furthermore, the previous studies primarily focused on the effect of individual-level factors. The neighborhood/community factors were largely overlooked. A multilevel analysis allows the simultaneous examination of within- and between-community variability in outcomes [22, 23] and of the extent to which between community variability is “explained” by individual and community-level factors. Studies using these approaches can usually provide the simultaneous effect of community characteristics and individual-level covariates on the health outcome [24, 25].

Therefore, the current study aimed to identify the individual and community level factors influencing the uptake of sufficient TT immunization among pregnant women in Ethiopia. The implication of the study was just to provide an insight to tackle the hindering factors of TT immunization in Ethiopia by providing the evidence for policymakers to enable them to strengthen currently ongoing immunization programs.

## Method

### Data source, study design and setting

This study utilized secondary data from the 2016 Ethiopian Demographic and Health Survey (EDHS). The data are analyzed using bivariate and multivariate multilevel logistic regression. The survey data were downloaded from the Measure DHS website after reasonable request and data use permission was fully guaranteed. The 2016 EDHS is part of the worldwide MEASURE DHS project which was funded by the United States Agency for International Development (USAID) and was implemented by the Ethiopian Central Statistical Agency. A DHS is undertaken every 5 years and the 2016 survey is the fourth Demographic and Health Survey in Ethiopia which covers all the nine regions and two administrative cities.

### Sample size and sampling procedure

The Ethiopian Demographic and Health Survey program (EDHS) has collected data on national representative samples of all age groups and key indicators including tetanus toxoid immunization among pregnant women. The information on the sociodemographic, socioeconomic, and maternal-related variables was also included in the survey.

**Fig. 1:**
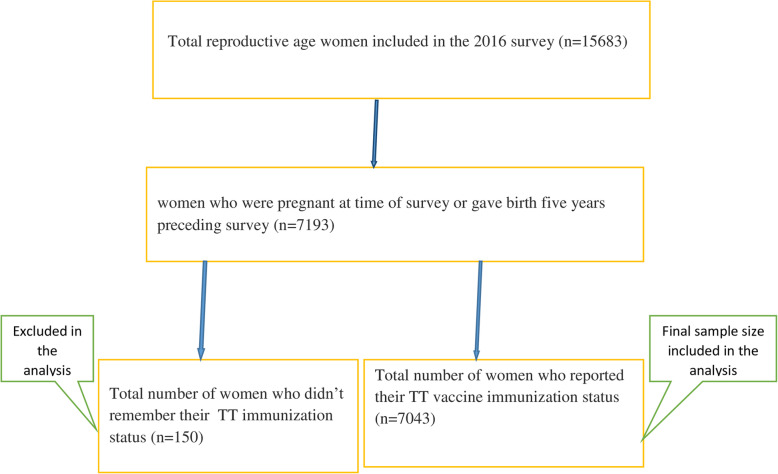
Data extraction procedure and sample size

A stratified two-stage cluster sampling procedure was employed to select study participants. In the 2016 survey, a total of 645 EAs (202 urban and 443 rural) were selected. From these enumeration areas, 18,008 households and from those households a total of 15,683 reproductive-age women were included in the survey. The relevant information on the sampling procedure and data quality can be accessed elsewhere [14]. For the current study, a total of 7043 pregnant women/who gave birth in five years preceding the survey were included (Fig. 1). The sampling weight was applied during the analysis to produce reliable estimates.

### Dependent variable

The dependent variable is defined as whether or not women received sufficient TT immunizations during their last pregnancy. A woman is considered to have sufficient TT if her immunization status corresponds to the WHO guidelines for the appropriate interval for protection at birth (PAB). According to this guideline, sufficient TT immunization is operationalized as receiving two or more doses during current pregnancy; or one dose during current pregnancy and at least one dose during before current pregnancy; or at least two TT doses before the current pregnancy of which the last dose was < 3 years before the birth; or three doses within the 5 years before the current pregnancy; or four doses with the last dose < 10 years before the pregnancy or receiving five doses or more before the current pregnancy [6]. Therefore, for the current study pregnant women who uptake two and above tetanus toxoid vaccine were considered as sufficiently immunized otherwise poorly immunized.

### Independent variables

From the 2016 EDHS dataset educational status of mother (no formal education, primary, secondary, higher) and husband (no formal education primary, secondary, higher), maternal age (15–20, > 20), maternal employment (not working, working), parity (primipara, multipara, grand multipara), wealth status (poor, middle, rich), the nature of pregnancy (wanted then, wanted later, no more wanted), antenatal care (ANC) visit (no visit, 2–3 visits, ≥4 visits), health care decision making (self, husband), health insurance (no, yes), perception of distance from the health facility (big problem, not a big problem), place of delivery (home, health facility), iron supplementation (yes, no), and media exposure (no, yes) were considered as individual-level variables.

The aggregate community level explanatory variables (community poverty level, community media exposure level, community illiteracy level, and community ANC utilization) were constructed by aggregating individual-level characteristics at the community (cluster) level. They were dichotomized as high or low based on the distribution of the proportion values computed for each community after checking the distribution by using the histogram. If the aggregate variable was normally distributed mean value and if not, normally distributed median value was used as a cut-off point for the categorization.

### Multilevel logistic regression analysis

Data Extraction, recoding, and both descriptive and analytical analysis were carried out using STATA version 14 software. Weighting was done to restore the representativeness of the sample so that the total sample looks like the country’s actual population. Descriptive analysis was conducted using cross-tabulation. As a result, frequencies and percentages were reported. The multilevel analysis was fitted due to the hierarchical nature of the demographic health survey data.

Consequently, four models were fitted. The first was the null model containing no exposure variables which was used to check variation in community and provide evidence to assess random effects at the community level. This model was used to assess the applicability of multi-level analysis by checking the random effects. The second model was the multivariable model adjustment for individual-level variables and model three was adjusted for community-level factors. In the fourth model, both individual and community-level variables were fitted with the outcome variable simultaneously. The regression equation is given as follows [26];

$$ \log \left[\frac{\varPi ij}{1-\varPi ij}\right]={\beta}_o+{\beta}_1{x}_{1 ij}+\dots +{\beta}_n{x}_{nij}+{u}_{oj}+{e}_{ij} $$

Where;

- πij is the probability of having poor TT uptake
- (1-πij) is the probability of good TT uptake
- *β*_*o*_ is log odds of the intercept
- *β*_1_, ... *β*_*n*_ are effect sizes of individual and community-level factors
- *x*_1*ij*_... *x*_*nij*_ are independent variables of individuals and communities
- The quantities *u*_*oj*_ and *e*_*ij*_ are random errors at cluster levels and individual levels respectively.

The odds ratio was used to estimate the association between the likelihood of poor TT immunization and explanatory variables at both community and individual levels and were expressed as odds ratios with a 95% confidence interval. Regarding the measures of variation (random-effects) intracluster correlation coefficient (ICC) and Proportional Change in Community Variance (PCV) were used.

## Results

### Sociodemographic characteristics of study participants

In this study, a total of 7043 pregnant women were included. Nearly two-thirds (66%) of study subjects had no formal education. The majority (70%) of respondents were unemployed and nearly half (50.39%)of them were poor. Regarding the place of delivery, about two-third (63%) of the participants were delivered at home. Moreover, 35.08 and 55% of participants did not receive ANC service and iron supplementation respectively. Most (96.54%) of respondents were not covered by health insurance had about 61% of respondents had no media exposure. Regarding the perception of distance to the health facility, nearly half (52%) of respondents perceived distance from the health facility as a big problem (Table 1).

**Table 1:** Sociodemographic characteristics of pregnant women in Ethiopia, 2016

Variables	Frequency	Percentage
Maternal age at first birth		
15–19	4298	61.03
> 20–34	2745	38.97
Maternal education		
No formal education	4277	60.73
Primary education	1899	26.96
Secondary education	565	8.02
Higher education	302	4.29
Maternal occupation		
Working	2108	29.93
Not working	4935	70.07
Wealth status		
Poor	3549	50.39
Middle	1007	14.30
Rich	2487	35.31
Health insurance coverage		
Yes	244	3.46
No	6799	96.54
Distance from the health facility		
Big problem	3721	52.83
Not big problem	3322	47.17
Media exposure		
Yes	2481	35.23
No	4562	64.77
Place of delivery		
Home	4421	62.77
Health facility	2622	37.23
Wanted pregnancy		
Then	5633	79.98
Later	964	13.69
No more	446	6.33
Husband education		
No	3129	44.43
Primary	2108	29.93
Secondary	733	10.41
Higher	1073	15.23
Health care decision		
Self	5248	80.40
husband	1279	19.60
ANC visit		
No visit	2471	35.08
One-three	2058	29.22
Four and above	2514	35.70
Iron supplementation		
No	3858	54.78
Yes	3185	45.22
Parity		
Primiparous	1442	20.47
Multiparous	3769	53.51
Grand multiparous	1832	26.01
Community poverty level		
Low	3592	51.00
High	3451	49.00
Community illiteracy level		
Low	3671	52.12
High	3372	47.88
Community ANC utilization		
Low	3556	50.49
High	3487	49.51
Place of residence		
Urban	1462	20.76
Rural	5581	79.24
Community media exposure		
Low	3546	50.35
High	3497	49.65

Regarding the community-level characteristics, the majority (79.24%) of participants were from rural communities and 47.88% of them were from communities with high illiteracy levels. Looking at community ANC utilization, nearly half (50.49%) of study subjects were from communities with high ANC utilization (Table 1).

### Random effect and model comparison

In the null model, variance component analysis was performed to decompose the total variance of poor TT immunization. The community-level variance which indicates the total variance of poor TT immunization that can be attributed to the context of the community in which the women were living was estimated. The applicability of multi-level analysis was justified by the significance of the community-level variance [community variance = 1.14; standard error (SE) = 0.11; *P*-value = 0.001], indicating the existence of significant differences between communities regarding poor TT vaccine utilization. This was further supported by the ICC in the null model which showed that about 26% of the variation of poor TT immunization among pregnant women was attributed to the difference at community-level factors. Besides, the higher PCV value (65%) in the final model indicates that about 65% of the variation of poor TT immunization among pregnant women was attributable to both the individual level and community-level factors. Regarding model comparison, we used deviance to assess model fitness. Consequently, the model with the lowest deviance value (Model IV) was found to be the best-fitted model (Table 2).

**Table 2:** Multilevel logistic regression analysis of factors associated with poor tetanus toxoid immunization among pregnant women in Ethiopia, 2016

Variables	Model I	Model II AOR 95% CI	Model III AOR 95% CI	Model IV AOR 95% CI
Maternal education				
No formal education	–	0.88(0.61,1.27)	–	0.91(0.64–1.30)
Primary education	–	0.80 (0.56–1.13)	–	0.86(0.61–1.20)
Secondary education	–	0.98 (0.69–1.39)	–	1.02(0.72–1.44)
Higher education	–	1.00	–	1.00
Maternal occupation				
Working	–	1.00	–	1.00
Not working	–	1.17(1.02–1.34)	–	1.15(1.10–1.31)^*^
Wealth status				
Poor	–	1.25(1.05–1.51)	–	1.26(1.03–1.54)^*^
Middle	–	1.12(0.91–1.37)	–	1.15(0.93–1.42)
Rich	–	1.00	–	1.00
Wanted pregnancy				
Then	–	1.00	–	1.00
Later	–	0.98(0.83–1.17)	–	0.99(0.83–1.18)
No more	–	0.97(0.75–1.26)	–	1.01(0.77–1.29)
Distance from the health facility				
Big problem	–	1.00	–	1.00
Not big problem	–	0.89(0.78–1.03)	–	0.89(0.78–1.03)
Media exposure				
Yes	–	1.00	–	1.00
No	–	1.32(1.13–1.53)	–	1.29(1.10–1.51)^**^
Husband education				
No	–	1.13(0.91–1.41)	–	1.13(0.90–1.41)
Primary	–	1.08(0.87–1.34)	–	1.11(0.89–1.38)
Secondary	–	0.99(0.77–1.26)	–	0.99(0.77–1.26)
Higher	–	1.00	–	1.00
Health care decision				
Self	–	1.00	–	1.00
Husband	–	1.01(0.87–1.18)	–	1.01(0.87–1.18)
Health insurance coverage				
Yes	–	1.31(0.94–1.82)	–	1.32(0.95–1.84)
No	–	1.00	–	1.00
Maternal age at first birth				
15–19	–	1.00	–	1.00
> 20	–	1.28(0.84–1.25)	–	1.24(0.83–1.21)
Place of delivery				
Home	–	1.00	–	1.00
Health facility	–	0.91(0.78–1.06)	–	0.91(0.78–1.06)
ANC visit				
No visit	–	5.94(2.76–7.61)	–	5.64(2.48–7.30)^***^
One-three	–	1.51(1.20–1.83)	–	1.50(1.19–1.82)***
Four and above	–	1.00	–	1.00
Iron supplementation				
Yes		0.59(.52–0.67)		0.59(0.51–0.68) ^***^
No	–	1.00	–	1.00
Parity				
Primiparous	–	1.00	–	1.00
Multiparous	–	1.05(0.90–1.24)	–	1.05(0.90–1.24)
Grand multiparous	–	0.99(0.80–1.21)	–	0.99(0.81–1.22)
Community ANC utilization				
Low	–	–	2.61(2.17–3.14)	1.03(0.84–1.27)
high	–	–	1.00	1.00
Residence				
Urban	–	–	1.00	1.00
Rural	–	–	1.08(0.87–1.34	1.13(1.08–1.72)^*^
Community media exposure				
Low	–	–	1.55(1.28–1.87)	1.17(0.94–1.44)
High	–	–	1.00	1.00
Community illiteracy level				
Low	–	–	1.00	1.00
High	–	–	1.58(1.31–1.91)	1.28(1.03–1.58) *
Community poverty level				
Low	–	–	1.00	1.00
High	–	–	1.14(0.94–1.39)	0.97(0.77–1.23)
Random effects				
Community variance (SE)	1.14(0.11)	0.51(0.07)	0.45(0.05)	0.40(0.03)
ICC (%)	26	17	13	11
PCV (%)	Reference	55.3	60.5	64.91
Model fitness				
Deviance	8961.60	6828.90	8608.71	6819.43

*AOR* Adjusted Odds Ratio, *CI* Confidence Interval; * = *P* < 0.05; ** = *P* < 0.01;*** = *P* < 0.001

### Factors associated with poor TT immunization

In the multilevel logistic regression analysis (model IV), antenatal care visit, iron supplementation, media exposure, maternal occupation, and wealth index were significantly associated with poor TT immunization. Regarding the community-level factors, place of residence and community illiteracy level had a significant association with poor TT immunization (*p* < 0.05).

The odds of poor TT immunization among pregnant women with no ANC visit and those who had one to three visits was 5.64[Adjusted odds ratio (AOR) = 5.64; 95% CI:2.48,7.30] and 1.50 (AOR = 1.49; 95% CI: 1.19–1.82) respectively times higher as compared to women who had more than three ANC visits. Looking to wealth status pregnant women from poor households had 26% (AOR) = 1.26; 95% CI: 1.03, 1.54) increased odds of poor TT immunization as compared to women from rich households. The odds of poor TT immunization was 41% (AOR = 0.77; 95% CI: 0.51, 0.68) lower among pregnant women who had received iron during pregnancy as compared to their counterparts. The odd of having poor TT immunization was 1.29 (AOR = 1.29; 95% CI: 1.10, 1.51) times higher among women who were exposed to media as compared to women who weren’t exposed to media. The likelihood of having poor TT immunization was 1.15 (AOR = 1.15; 95% CI: 1.10, 1.31) times higher among unemployed women as compared to their counterparts.

Regarding the place of residence, the odds of poor TT immunization among women from rural communities was 1.13 (AOR = 1.13; 95% CI: 1.08, 1.72) times higher as compared to women who live in urban communities. Moreover, pregnant women from communities with higher illiteracy had 28% (AOR = 1.28; 95% CI: 1.03, 1.58) higher odds of poor TT immunization as compared to their counterparts (Table 2).

## Discussion

This study examined the association between several demographic, socioeconomic, and health care access-related factors with poor tetanus toxoid (TT) immunization during pregnancy.

Although the proportion of pregnant women receiving 4 or more antenatal care (ANC) visits has no necessary relationship with the actual tetanus toxoid vaccine uptake, it is used prominently as a global benchmark indicator to track maternal health program performance [27]. Thus, the odds of poor TT immunization among pregnant women who didn’t have antenatal care visit was strongly higher than those who have at least four visits. This result is similar to findings in other studies [17, 18]. Thus, strategies aimed at improving antenatal care attendance among pregnant women would be a step in the right direction towards increasing TT immunization. Likewise, antenatal care providers should encourage women and provide information about the importance of getting immunized since ANC follow-up provides an opportunity to convey such a critical message to pregnant women so as to increase TT uptake.

Although global poverty has fallen dramatically over the past decades, it has still pronouncing effect on inequality in health service utilization especially in developing countries [28]. The asset indices are used as diagnostic tools for private household wealth and basic public goods access for better health service utilization [29]. Thus the current study highlighted the effect of the household wealth index on the uptake of the TT vaccine. Therefore, women from households with poor household wealth index had higher odds of poor TT immunization as compared to those from rich households. This is supported by the finding in Bangladesh [19]. This might be because the economic status is one of the prominent determinants of maternal healthcare-seeking behavior [30–32] possibly this will lead to low maternal health service utilization including immunization for those women from poor households. Moreover, the availability of free or subsidized services is known to improve the uptake of maternal services among the poor [32, 33].

Similarly, the likelihood of poor tetanus toxoid immunization was higher among unemployed mothers as compared to their counterparts. This finding is consistent with the finding in Bangladesh [19]. The possible justification could be an employed woman might involve in social communication which could create a context for learning and engagement in health-related dialogues, particularly during pregnancy. As a result, the workplace might operate as social support and motivational venue enhancing the utilization of health care services. Besides, The previous evidence indicates that formal employment is associated with a range of social outcomes and behaviors that are typically associated with higher levels of social development [34].

Consistent with studies in Eastern Ethiopia [21] and Bangladesh [19] the current study identified that the odds of TT vaccine uptake was lower among rural women as compared to those from urban communities. In the rural communities, most of the time TT immunization was provided through outreach and there is a lack of awareness among women to continue taking the doses even after delivery especially in remote areas of Ethiopia [35]. This is further supported by the evidence in the current study where more than half of participants perceived the distance from health facilities as a big problem (Table 1).

Though it doesn’t show a significant effect on TT immunization in India [36], media exposure is another important factor associated with tetanus toxoid utilization among pregnant women in the current study. Thus, the likelihood of poor TT immunization among non-exposed mothers was higher as compared to those who are exposed to media. The health service-related messages including immunization are commonly conveyed through media by the ministry of health and other stakeholders which builds their knowledge of TT immunization. Since knowledge of TT immunization is an important determinant of TT vaccine uptake in Ethiopia [20], those women who weren’t exposed to media may miss this opportunity.

The particular interest in this study is the effect of iron supplementation on tetanus toxoid vaccine utilization. This study indicated that the odds of poor TT immunization was lower in those mothers who were supplemented with an iron tablet during pregnancy as compared to their counterparts. Iron supplementation was a routine service during pregnancy like the TT vaccine in Ethiopia. Therefore, those mothers who received iron could have a high probability of getting TT immunization.

Moreover, in our study higher community illiteracy level was another important factor that was associated with higher odds of having poor TT immunization. Another study also revealed that maternal health service utilization is associated with literacy level in the community in which mothers from communities with higher illiteracy levels had a lower chance of utilizing maternal health services [37].

In general maternal and neonatal tetanus is considered to be a public health problem in Subsaharan Africa. Interventions to improve vaccination outcomes are commonly grouped into those targeting health services delivery such as improving human resources training, logistics, cold chain maintenance, and vaccine storage, effective financing, monitoring and evaluation, and supportive supervision. The most recent review considered shows that much remains to be done to improve immunization coverage among women in Subsaharan Africa [38]. Besides, identifying potential hindering factors of TT uptake is vital for policymakers. Thus, the findings in this study could have positive implications to improve the coverage of TT immunization among pregnant women and then reduce related maternal and neonatal adverse outcomes in Ethiopia.

The main strength of this study was the use of nationally representative data which will provide good evidence for policymakers at the national level. The other strength is the use of an appropriate model (multilevel) for analyzing the data. However, this study is not free of limitations. First, the cross-sectional nature of the study leads to difficulty to show the temporal relationship between the factors and the outcome. Second, immunization status was primarily collected through self-report making it susceptible to recall bias. Besides, variables such as lack of knowledge on the immunization schedule, the attitude of women towards TT immunization, level of community mobilization interventions, and other health care provider-related factors were not assessed owing to the secondary data analysis.

## Conclusion

In this study, both individual and community-level factors were associated with poor TT immunization among pregnant women. Accordingly, antenatal care (ANC) visits, media exposure, employment, place of residence, iron supplementation, and community illiteracy had a significant statistical association with poor TT immunization.

Therefore, focusing on antenatal care services especially encouraging pregnant women to have at least four visits, consulting women to be exposed to media, improving community literacy and maternal employment will help to minimize poor TT immunization. Besides, this study particularly focused on pregnant women. Thus it provides new evidence about the influence of socioeconomic factors on inequalities in the maternal health sector. This has implications for secondary policy priorities, such as the need to address uneven fertility transitions in sub-Saharan Africa and their wider interaction with poverty reduction efforts at the individual and community-level in developing countries [39].

## Data Availability

All relevant data are available within the manuscript.

## Abbreviations

ANC: Antenatal Care
AOR: Adjusted Odds Ratio
CSA: Central Statistical Agency
EAs: Enumeration Areas
EDHS: Ethiopia Demographic and Health Survey
MNTE: Maternal and Neonatal Tetanus Elimination
SNNP: South Nation Nationality and People
TT: Tetanus Toxoid
WHO: World Health Organization
